# Bacterial Quality, Prevalence of Pathogens, and Molecular Characterization of Biofilm-Producing *Staphylococcus aureus* from Korean Dairy Farm Environments

**DOI:** 10.3390/ani11051306

**Published:** 2021-04-30

**Authors:** Sangdon Ryu, Minhye Shin, Bohyun Yun, Woongji Lee, Hyejin Choi, Minkyoung Kang, Sangnam Oh, Younghoon Kim

**Affiliations:** 1 Department of Agricultural Biotechnology and Research Institute of Agriculture and Life Science, Seoul National University, Seoul 08826, Korea; ryusd@snu.ac.kr (S.R.); mhshin1984@snu.ac.kr (M.S.); tjrhrwnd@snu.ac.kr (W.L.); zni901@snu.ac.kr (H.C.); 2 Department of Functional Food and Biotechnology, Jeonju University, Jeonju 55069, Korea; boding33@gmail.com (B.Y.); mink118283@jj.ac.kr (M.K.)

**Keywords:** dairy farm environment, foodborne pathogens, *Staphylococcus aureus*, RNA-seq, biofilm, microbial contamination

## Abstract

**Simple Summary:**

In this study, we analyzed hygienic indicator bacteria and pathogenic microorganisms (*Salmonella* spp., *Escherichia coli* O157:H7, *Listeria monocytogenes*, *Bacillus cereus*, *Staphylococcus aureus*, *Clostridium perfringens*, *Campylobacter jejuni*/coli) in Korean dairy farms. As a result, *B. cereus* and *S. aureus* were detected in dairy farm environment. Total aerobic bacteria, psychrotrophic bacteria, coliform, and yeasts/molds differed slightly between dairy farms, but a few spots, such as floors, drain holes, and niches, showed high microbial counts in most of dairy farms. Subsequently, we performed RNA-seq analysis on *Staphylococcus aureus* JDFM SA01 isolated from a milk filter to determine the biofilm formation ability and characteristics. In biofilm, the significant up-regulation of genes encoding surface proteins and genes, which advance the adhesion, might clarify the increased biofilm viability and biomass. Therefore, in this study, spots with high possibility of microbial contamination could be identified in dairy farms and the basis for producing safe milk and dairy products by effective hygiene management against microbial contamination was established.

**Abstract:**

Raw milk acts as a mediator of major foodborne pathogenic bacterial infections. However, the sources of pathogens that contaminate milk are often unclear. This study assessed the prevalence of sanitary quality-indicating bacteria (total aerobic bacteria, psychrotrophic bacteria, coliform, and yeast/molds), including seven foodborne pathogens, in a dairy farm environment and processing plant in Korea. The microbiological analysis showed that a few sites, such as vat bottoms, room floors, drain holes, and niches, showed high microbial loads in most dairy farms. Based on quantitative microbial tests, *Bacillus cereus* was detected in three farms and *Staphylococcus aureus* was detected in only one farm. Among them, *S. aureus* JDFM SA01 isolated from a milk filter showed strong biofilm formation and toxicity to the host *Caenorhabditis elegans*. Subsequently, RNA-seq was performed to characterize the biofilm formation ability of *S. aureus* JDFM SA01. In biofilms, the significant upregulation of genes encoding microbial surface components and recognizing adhesive matrix molecules promotes adhesion might explain the increased viability and biomass of biofilms. This study provided insight into the prevalence of pathogenic bacteria and microbial contamination levels across dairy farms.

## 1. Introduction

Milk contains major nutrients that are essential for human health and is thus called a single balanced food because it contains high-quality protein, fat, lactose, vitamins, and minerals [1]. The consumption of milk and dairy products has long been associated with good health, but it can also pose a potential health hazard if mishandled or if the conditions of manufacture are not sufficiently hygienic and safe [2].

Milk provides a suitable environment for many microorganisms due to its high water content and essential nutrient availability. Bacteria, yeasts, and molds are common contaminants in milk [3], and some microbes promote the spoilage of milk [4] and degrade milk and dairy products. As such, the presence and growth of microorganisms in milk significantly impact the quality of milk [5] and can harm the dairy industry and public health. The main pathways for raw milk contamination are direct contact with contaminated sources in the dairy environment (e.g., soil, feces, feed, water, air, milking equipment, and sick animals) and secretions from the udders of infected animals [6].

Dairy farms have a very complex microbial ecology and a variety of complicated environments. Therefore, various pathogenic microorganisms exist in the dairy environment [7]. In most cases, pathogens are inactivated during the pasteurization process. However, pasteurization of raw milk does not eliminate contamination of the milk or dairy products by pathogens in the postprocessed products in dairy processing plants [6]. Such contamination may cause food poisoning in consumers.

*S. aureus* can produce a wide variety of enterotoxins, and staphylococcal food poisoning has caused many infections worldwide, with symptoms of diarrhea, nausea, and abdominal cramps [8,9]. Staphylococcal food poisoning is related to the contamination of *S. aureus* after pasteurization or to a large amount of toxin produced by the organism before pasteurization. *S. aureus* can persist by forming biofilms in various environments, such as the host and food processing surfaces, avoiding host defenses and antimicrobial agents [10]. This situation can create ideal conditions for *S. aureus* proliferation and lead to colonization and biofilm formation on surfaces [11,12].

In this study, we collected samples from 11 dairy farms located in Korea, focusing on the dairy environment and processing plants. The collected samples were used to investigate the prevalence of microorganisms in the dairy environment and processing plants that primarily influence the quality and stability of milk and dairy products. In addition, we identified the toxicity and biofilm formation ability of *S. aureus* JDFM SA01 isolated from a milk filter and previously subjected to whole-genome sequence analysis [13]. Further analysis of the transcriptomes of *S. aureus* JDFM SA01 biofilms and planktonic cultures verified the expression of virulence genes associated with biofilm formation.

## 2. Materials and Methods

### 2.1. Collection of Dairy Farm Samples

A total of 11 farms located throughout South Korea were included in this survey. These comprised seven dairy farms (farms A, B, C, D, E, F, and G) of Jeolla Province, one dairy farm (H) of Gyeonggi Province, one dairy farm (I) of Chungcheong Province, and two dairy farms (J and K) of Gangwon Province. None of the farms in this study have not experienced any hygiene problems.

A total of 444 samples were collected, consisting of 171 samples from a dairy farm environment and 273 samples from a processing plant (Table 1). The samples were from 6 sites within the dairy farm environment and 10 sites within the processing plant. The raw milk, cheese, and drinking water were collected in a sterilized pack at 25 g/mL, and milk filters were aseptically cut into 30–50 cm^2^ pieces [14]. The other samples were rubbed with a Swab kit (3M Quick Swab, USA) in a 10 cm × 10 cm area. Samples were stored at 4 °C until analysis, and they were analyzed within 24 h of collection.

### 2.2. Microbial Analysis of the Dairy Farm Environment and Processing Plants

#### 2.2.1. Measurement of Hygiene-Indicating Microorganisms

The raw milk, cheese, and drinking water collected for quantitative analysis were added at 25 g/mL to 225 mL of 0.1% peptone water (PW, Oxoid, Hampshire, UK) in a stomacher bag, homogenized for 2 min with a stomacher (Bagmixer 400VW, Interscience^®^, Paris, France), diluted 10-fold, and used for analysis. The milk filter pieces were weighed in a filtered stomacher bag, diluted 1:9 (*wt/wt*) with 0.1% peptone water, and pummeled in a stomacher for 2 min. In addition, samples collected with the swab kit were diluted 10-fold without any pretreatment to analyze microorganisms.

One milliliter of the pretreated sample was diluted in 9 mL of sterilized 0.1% peptone water in 10-fold steps, and 1 mL sample was taken at each dilution concentration and dispensed into 3M dry film medium to analyze total aerobic bacteria, psychrotrophic bacteria, coliform, yeasts, and molds. According to the manufacturer’s instructions, subsequent triplicate spread plating was performed on Petrifilm^TM^ aerobic plate count (APC) plates, Petrifilm^TM^ coliform count plates, and Petrifilm^TM^ yeast and mold count plates. APCs and coliform plates were incubated aerobically at 37 °C for 24 h, and psychrotrophic bacteria were incubated on APC plates aerobically at 25 °C for 48 h. Yeast and mold plates were incubated aerobically at 25 °C for 72 h in an aerobic incubation chamber. Counts were recorded as colony forming units per gram (CFU/g).

#### 2.2.2. Detection and Identification of Foodborne Pathogens

Each sample was investigated for contamination with major foodborne pathogens, *E. coli* O157:H7, *L. monocytogenes*, *Salmonella* spp., *S. aureus*, *Campylobacter jejuni/coli*, *C. perfringens*, and *B. cereus*. Pathogenic bacteria were inoculated into different pathogenic bacteria selection media after the enrichment process, and qualitative analysis was performed through separation culture and identification experiments. Each foodborne pathogen was analyzed according to the Food Code of the Ministry of Food and Drug Safety of Korea [15].

### 2.3. Caenorhabditis Elegans Life Span Assay

To estimate how *S. aureus* JDFM SA01 affects the host life span, the *C. elegans* life span was determined using a slight modified method [16]. Briefly, 100-μL aliquots of concentrated bacteria (*S. aureus* JDFM SA01, *S. aureus* Newman as a control) were exposed to NGM plates and live worms were counted daily. To achieve exact counts, *C. elegans* were transferred to new plates containing bacteria every 3 days. All worms were incubated at 25 °C, and they were regarded dead when they did not react to a light touch.

### 2.4. Biofilm Formation and Sample Collection

*S. aureus* JDFM SA01 and RN 4220 (5 × 10^7^ CFU/mL) were inoculated into 0.5× and 0.1× LB broth on 96-well polystyrene plates at 37 °C for 5 days. To quantify biofilm formation, planktonic bacteria were removed by gentle washing three times with phosphate-buffered saline (PBS) and biofilms were stained with 200 µL 0.1% crystal violet for 20 min at room temperature. Biofilms were dissolved in 200 µL 95% ethanol solution, and absorbance was measured at 550 nm in a spectrophotometer (SpectraMax ABS Plus, Molecular Devices, San Jose, CA, USA) [17].

Extraction of RNA from the *S. aureus* JDFM SA01 biofilm culture was performed following the previously established method [18]. Glass wool (0.5 g) was added to 100 mL of LB broth, supplemented with 0.1% (*w/v*) glucose, and the preculture was inoculated with 1 mL (1:100 dilution). After incubation for 24 h at 37 °C and shaking at 200 rpm, the glass wool was rinsed three times in 0.85% NaCl. The cells were shaken vigorously for 30 s to detach the bacterial biofilms from the glass wool surface in sterile saline (0.85% solution of sodium chloride). After that, the cells were disrupted by sonication (10 s on/10 s off, 60 cycles). Bacteria were harvested by centrifugation at 5000× *g* for 10 min at 4 °C.

### 2.5. RNA Sequencing

#### 2.5.1. RNA Extraction, Library Construction, and Sequencing

Total RNA was extracted using TRIzol reagent (Invitrogen, Carlsbad, CA, USA) and an RNeasy Mini kit (QIAGEN, Valencia, CA, USA) based on the manufacturer’s instructions. The concentration (260/280 ratio and 260/230 ratio) and quality of total RNA were determined using a spectrophotometer (SpectraMax ABS Plus, Molecular Devices, San Jose, CA, USA). For RNA-seq, a TruSeq RNA Sample Prep Kit v2 (Illumina, San Diego, CA, USA) was used according to the manual, and the cDNA library was generated according to the basic protocol provided by Illumina. Libraries were then sequenced on an Illumina HiSeq 2000 platform with paired-end read sequencing (2 × 150 bp).

#### 2.5.2. RNA Sequencing Data Analysis

The adapter sequence was removed from raw reads using Trimmomatic 0.38 [19] bases, with base quality less than 3 from the ends of the reads, and bases not satisfying the window size = 4 and mean quality = 15 were removed with the sliding window trim technique. After that, trimmed data were generated, with reads shorter than 36 bp removed, and further analysis was performed based on high-quality reads. The index of the reference genome was constructed using the Hisat2 v2.1.0 program (https://daehwankimlab.github.io/hisat2/ accessed on 4 November 2020) [20], and paired-end clean reads of the *S. aureus* subsp were read and compared. Next, uniquely mapped reads were quantified with Subread/featureCounts version v1.5.1 (http://subread.sourceforge.net/ accessed on 4 November 2020) [21], using ENSEMBL version 82 transcriptome definitions. The generated data were subjected to differential expression analysis between various types of samples using the R package edgeR [22]. The threshold value |log2-fold change > 1| and *p*-value < 0.05 were used to define genes as significantly differentially expressed.

To identify the function of differentially expressed genes, Gene Ontology (GO) annotations were analyzed using the DAVID online tool [23] and clusterProfiler [24]. Analysis was performed on selected DEGs and analyzed with a focus on gene functional annotations of cellular components (CC), biological processes (BP), and molecular functions (MF). The Kyoto Encyclopedia of Genes and Genomes (KEGG) pathway [25] annotations and enrichment analysis were conducted using the Search Tool for the Retrieval of Interacting Genes/Proteins v11.0 (https://string-db.org accessed on 25 November 2020) (STRING) with FDR-adjusted *p*-value ≤ 0.05 [26].

## 3. Results

### 3.1. Microbial Quality of Farm Environment and Processing Plant

#### 3.1.1. Total Aerobic Bacteria

Total aerobic bacteria counts revealed a high level of contamination in the farm environment as expected, and the highest number of bacteria was found in soil and feces, with 6.6~9.5 log CFU/g. The contamination level of drinking water was 3.2~5.8 log CFU/mL, and that of worker shoes was 3.4~8.8 log CFU/100 cm^2^. In a milking station, the bottom of the milking station was the most contaminated site at the level of 2.1~9.0 log CFU/100 cm^2^, and that of the milk filter was confirmed to be 3.3~9.2 log CFU/100 cm^2^. The udder junction of the milking machine was found to have a level of 1.3~4.5 log CFU/100 cm^2^, except in areas that microorganisms did not contact (Table 2).

In the processing plant, the total aerobic bacteria count was confirmed to be lower than that in the farm environment (Table 3). The inside of the cheese vat, cheese knives, and cheese molds that are in direct contact with the cheese were managed hygienically on most farms, but three farms detected bacteria at a level of 0.5~4.2 log CFU/100 cm^2^ inside the cheese vat. In addition, at the bottom of the cheese vat, which was not in direct contact with cheese, the level was 2.1~8.3 log CFU/100 cm^2^. In the processing room floor, the drain hole, and the niche, the contamination was found to be 0.7~6.4 log CFU/100 cm^2^, 2.0~7.5 log CFU/100 cm^2^, and 1.9~5.8 log CFU/100 cm^2^, respectively. In the ripening room, the space for ripening cheese, the floor of the ripening room, table, and under the ripening table, bacterial counts were 0.5~5.5 log CFU/100 cm^2^, 0.7~4.6 log CFU/100 cm^2^, and 0.7~6.9 log CFU/100 cm^2^, respectively. The count was lower in the ripening room than in the processing room producing cheese. In raw milk and cheese, the bacterial counts were 4.1~6.2 log CFU/mL and 1.8~8.0 log CFU/g, respectively.

#### 3.1.2. Psychrotrophic Bacteria

The psychrotrophic bacteria were present at similar levels as total aerobic bacteria (Table 4). They showed the highest contamination levels in soil and feces; 5.1~9.2 log CFU/g in the farm environment and, in drinking water, 2.7~7.4 log CFU/mL, except on one farm. The worker shoes had 1.6~6.0 log CFU/100 cm^2^ bacterial count, and the udder junction of the milking machine had 2.1~4.1 log CFU/100 cm^2^, except in four farms where bacteria were not detected. In the milk filter, the number of bacteria was 3.6~7.1 log CFU/100 cm^2^ except in two farms.

In the processing plant, psychrotrophic bacteria were not detected inside most cheese vat, cheese knife, or cheese mold sites but were detected in one farm inside the vat at 2.2±0.1 log CFU/100 cm^2^ and in the molding frame in one farm at 4.1 ± 0.6 log CFU/100 cm^2^. The bottom of the cheese vat had bacteria at 1.5~7.2 log CFU/100 cm^2^, and the vat bottom, drain hole, and niche of the processing plant had contamination at 0.7~5.2 log CFU/100 cm^2^, 1.6~5.8 log CFU/100 cm^2^, and 2.2~5.8 log CFU/100 cm^2^, respectively (Table 5). The psychrotrophic bacteria count of each location showed a similar tendency to that of most total aerobic bacteria.

#### 3.1.3. Yeasts and Molds

The yeasts and molds showed the highest contamination level in soil and feces at a level of 2.3~6.5 log CFU/g among dairy farm environments. Next, the contamination was high, on the order of 1.5~5.1 log CFU/100 cm^2^ on the worker shoes in contact with the soil and feces and 2.3~4.8 log CFU/100 cm^2^ on the milking floor (Table 6).

Yeasts and molds were also detected in various sites in the processing plant. In particular, in the vat insides, cheese knife, and cheese mold, the number of yeasts and molds were 0.5–3.4 log CFU/100 cm^2^, 0.5–3.4 log CFU/100 cm^2^, and 0.5–3.4 log CFU/100 cm^2^, respectively (Table 7). The degree of contamination was highest in the vat bottom, niche, and drain hole.

#### 3.1.4. Coliform Count

Coliforms (*Escherichia, Klebsiella, Enterobacter,* and *Citrobacter* belong to the coliform group), which are pollution-indicative bacteria, showed the highest contamination level of 3.8~6.2 log CFU/g, excluding five farms where these bacteria were not detected in soil and feces (Table 8). Coliform counts in the drinking water and the worker shoes were 1.5~2.8 log CFU/mL and 1.6~4.5 log CFU/100 cm^2^, respectively. These bacteria were not detected in the udder junction of the milking machine in most farms, but they were detected at a level of 1.5 ± 0.5 log CFU/100 cm^2^ in one farm, showing a low level of contamination.

In the processing plant, coliforms were rarely detected in the cheese vat, cheese knives, and cheese molds but existed at the level of 3.4 ± 0.0 log CFU/100 cm^2^ inside cheese vats in of one farm and detected at 3.2 ± 0.2 log CFU/100 cm^2^ in the cheese molds in another farm (Table 9). In addition, it was detected in the various sites within the processing plant, such as the processing plant floor, drain hole, niche, ripening room floor, and ripening table bottom. In raw milk and cheese, the contamination was 0.8~3.8 log CFU/mL and 1.0~3.2 log CFU/g, respectively.

### 3.2. Detection of Foodborne Pathogens in Farm Environments and Processing Plants

The qualitative test results of the samples (171) collected from 11 farm environments showed the positive detection of the *B. cereus* group and *S. aureus* in 8.2% and 1.8% of samples, respectively. The highest portion of the detected pathogens was the *B. cereus* group in soil and feces, present in 18.5% of the samples tested, followed by boots (18.2%) and milking floors (11.1%). Interestingly, when the milk filter was tested in addition to the raw milk sample, the detection probability increased, suggesting that a portion of milk contamination could occur during the process of filtering out substances, such as dust, manure, straw, or insects. Previous studies have also detected various pathogenic bacteria in the milk filter [14,27,28]. During pathogen detection, in the raw milk sample and the milk filter, three cases of *S. aureus* were positive only in the milk filter. The others (*E. coli* O157, *L. monocytogenes*, *C. perfringens*, *Salmonella* spp., and *Campylobacter* spp.) were not detected in the farm environment (Table 10 and Table 11).

### 3.3. Toxicity and Biofilm Formation of S. aureus JDFM SA01

**Figure 1:**
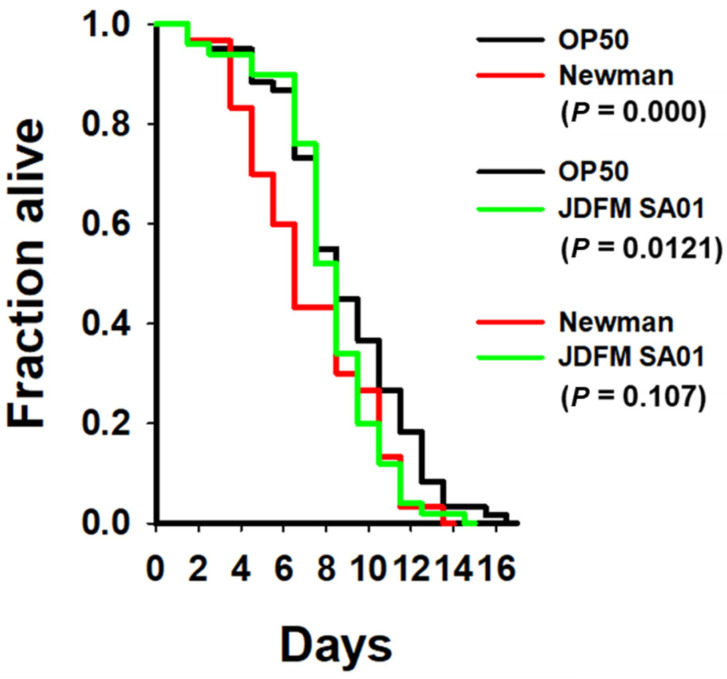
The survival rate of *C. elegans* worms infected with *S. aureus* JDFM SA01. Survival statistics: *S. aureus* JDFM SA01-conditioned nematodes compared with worms feeding on *S. aureus* Newman and OP50 control strains. Statistical analysis was performed using Kaplan-Meier method.

During the process of assessing microbial quality and prevalence of pathogens, we isolated a *S. aureus* strain (JDFM SA01) from the milk filter in a farm and performed whole-genome sequence analysis of the strain [13]. In order to further investigate its molecular characteristics, we analyzed its pathogenicity-related characteristics by toxicity tests and biofilm formation analysis. Consequently, we first studied the lifespan of *C. elegans* to confirm the toxicity of *S. aureus* JDFM SA01. As a control, we used *S. aureus* Newman, which is generally recognized as a robust virulence phenotype. We found that the life span of *C. elegans* exposed to *S. aureus* JDFM SA01 was not significantly different from that of *C. elegans* exposed to *S. aureus* Newman (Figure 1).

**Figure 2:**
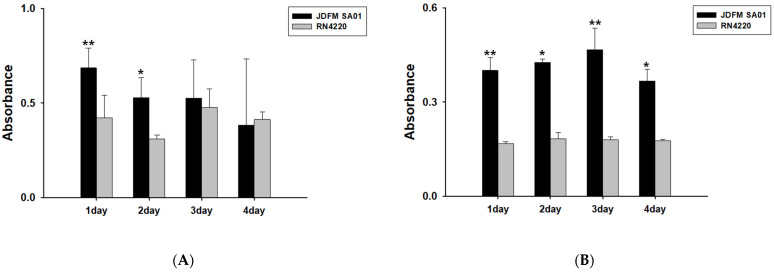
Evaluation of the biofilm formation ability of *S. aureus* JDFM SA01 strains. (**A**) 0.5× LB broth, (**B**) 0.1× LB broth. Statistical significance was analyzed with a *t*-test. Asterisks indicate a statistically significant difference compared with RN4220 (* *p* < 0.05, ** *p* < 0.01).

The milk filter is in direct contact with the milking machine. Therefore, *S. aureus* JDFM SA01 isolated from milk filters may be a contaminant in various facility sites, including milking machines, milking pipes, and bulk tanks, and can continuously contaminate raw milk by biofilm formation. Therefore, to confirm the biofilm-forming ability of *S. aureus* JDFM SA01, it was compared with the well-characterized biofilm-forming reference strain *S. aureus* RN4220. The *S. aureus* JDFM SA01 strain showed higher biofilm formation ability on average than *S. aureus* RN4220 in 0.5 X and 0.1 X LB broth (Figure 2A,B). Our results showed the high toxicity and biofilm formation ability of *S. aureus* JDFM SA01. These results indicate the possibility of contamination of milking equipment with *S. aureus* and suggest continuous contamination of raw milk through biofilm formation when contaminated.

### 3.4. Transcriptome Analysis Overview

To study the gene expression profiles in biofilms, transcriptomics analysis of biofilms and planktonic cells was performed by RNA sequencing. Quality control analysis of raw reads obtained through sequencing was conducted, and basic statistics, such as total read quality, total bases, total reads, and GC (%), were produced. The total number of sequenced bases of planktonic cells was 2,967,007,955, and the number of reads was 29,473,682, of which 96.50% showed a Phred score of 30. For biofilms, a total of 3,115,678,013 sequenced bases were identified, and 30,942,842 reads were identified, of which 96.48% had a Phred score of 30. To reduce the bias of the analysis result, all samples were pretreated to remove artifacts such as adapter sequences, contaminant DNA, and PCR duplicates.

The cDNA fragment obtained through RNA-seq was mapped using a genomic DNA reference. Among 29,473,682 reads of planktonic cells, 28,548,736 reads were mapped, the remaining 860,642 reads were not mapped, and 64,304 reads were removed due to multiple mapping. In biofilms, 30,074,628 reads out of 30,942,842 reads were mapped. The remaining 822,706 reads were not mapped, and 45,508 reads were removed due to multiple mapping. To check the data quality of known genes from read mapping, genes with a count value of 0 in more than one sample were excluded from the analysis. A total of 51 genes were excluded from planktonic cells, and 73 genes were excluded from biofilms. After the statistical analysis was performed on 2506 genes, excluding 124 out of 2630 genes, differentially expressed genes were analyzed.

### 3.5. Quantitative Analysis of Gene Expression after Treatment of S. aureus JDFM SA01 Biofilms

**Figure 3:**
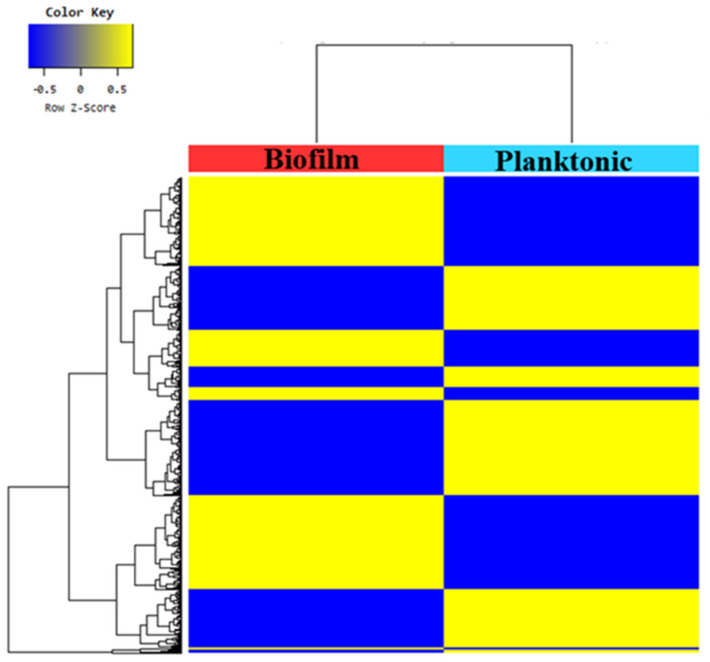
Heat map of the one-way hierarchical clustering using Z-score for normalized value (log2 based).

A heat map of hierarchical clustering analysis (Euclidean distance, complete linkage) shows gene expression patterns between the two samples (Figure 3), divided into cluster groups, showing a total of 10 expression pattern changes. In total, 501 upregulated genes and 489 downregulated genes with significant differences between the two samples were analyzed.

**Figure 4:**
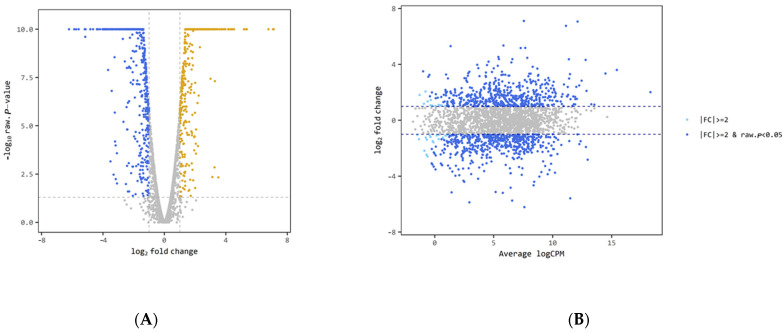
Differential expression level of the biofilm vs. planktonic identified by | log2 (fold change)| > 1 and adjusted *p*-value < 0.05. (**A**) volcano plot, (**B**) smear plot.

Next, a volcano plot and smear plot were constructed to visualize how the differentially expressed genes (DEGs) were distributed. The results showed that genes with different levels of expression existed between the two groups (Figure 4).

*S. aureus* JDFM SA01 had numerous genes encoding microbial surface components, recognizing adhesive matrix molecules (MSCRAMMs), including fibronectin-binding protein A precursor (fnbA), fibrinogen-binding protein (fib), collagen adhesin (cna), putative poly-beta-1,6-N-acetyl-D-glucosamine export protein (icaC), and iron-regulated surface determinant protein (isdA, isdB, isdC, isdH). Genes associated with the immune evasion cluster (IEC) encoding staphylococcal complement inhibitor (scn) were expressed more in biofilms than in planktonic cells. Notably, the virulence genes hlgA and hlgC were overexpressed in biofilms (Table 12).

### 3.6. GO Functional Enrichment Analysis

**Figure 5:**
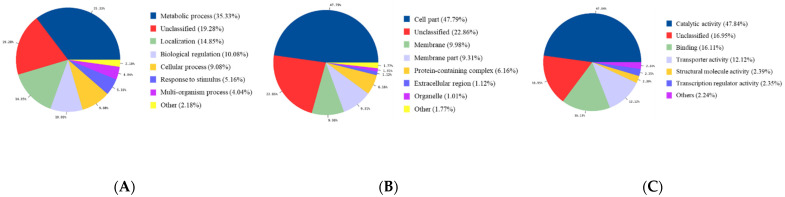
Significantly enriched gene ontology terms of differentially expressed genes. (**A**) Biological process; (**B**) cellular component; (**C**) molecular function.

To further analyze the function of the DEGs underlying the difference between planktonic and biofilms, GO enrichment analysis was performed with 990 DEGs. The details regarding GO terms (biological process, cellular component, and molecular function) were related to biofilm and planktonic states. In the category of biological process, metabolic process was 35.33%, localization was 14.85%, biological regulation was 10.08%, the cellular process was 9.08%, the response to the stimulus was 5.16%, and the multi-organism process was 4.04% (Figure 5A). In the category of molecular function, catalytic activity was 47.84%, binding was 16.11%, transporter activity was 12.12%, structural molecule activity was 2.39%, and transcription regulator activity was 2.35% (Figure 5B). In the category of cellular component, cell part was 47.79%, membrane part was 9.98%, membrane part was 9.31%, protein-containing complex was 6.16%, extracellular region was 1.12%, and organelle was 1.81% (Figure 5C).

## 4. Discussion

This study found that many farms have been hygienically managed, whereas some farms exhibited high levels of contamination. In particular, the milking machine needs thorough hygiene management because milk can be contaminated with pathogenic bacteria during milking [7]. However, in the farm environment, the udder junction of the milking machine showed total aerobic bacteria counts of up to 4.5 log CFU/100 cm^2^. In the processing plant, high levels of total aerobic bacteria were detected at the bottom of the cheese vat, the drain hole, and the bottom of the ripening table. Even though the three sites mentioned above do not come into contact with the cheese, they may be exposed to contamination during cheese production, so care should be taken against microbial contamination. The vat inside one processing plant showed microbial contamination of approximately 4.2 log CFU/cm, suggesting the need for thorough washing.

Total aerobic bacteria counts are basic and good hygiene indicators for evaluating the degree of microbial contamination and the general quality of milk and dairy products [29,30]. Our results showed large variations in the total aerobic bacterial prevalence among the tested spots on different farms and that there were different bacterial counts in each spot. In particular, some farms are considered hygienically managed even inside the milking machine and vat inside, whereas some farms would need strict hygiene management as a high level of contamination was detected.

Psychrotrophic bacteria are currently thought to be one of the main troubles related to the microbial contamination of raw milk [31]. It is known that psychrotrophic bacteria present in milk are closely related to the spoilage of milk and dairy products and, as a result, have a direct impact on the transport and shelf life of milk [32]. Several genera exist in psychrotrophic bacteria, and *Pseudomonas* and *Bacillus* are considered the major genera in dairy [33]. *Pseudomonas* is considered a psychrotrophic bacterium, with active metabolic activity and proliferation at 4–7 °C. In our study, the number of total aerobic bacteria and psychrotrophic bacteria showed a similar trend of prevalence. This finding suggests that the majority of the bacteria present in the dairy environment are psychrotrophic bacteria, and milk can be contaminated by these bacteria at any time. Therefore, it is essential to manage farm hygiene to prevent the possibility of milk contamination from these psychrotrophic bacteria.

The presence of coliform above 2.0 log CFU/mL indicates a hygienically inappropriate environment for milk production [34]. Coliforms exist extensively in dairy environments, including soils, rivers, groundwater, milking machines, and feces, and can easily contaminate the raw milk [35]. In our study, coliforms of a minimum of 1.5 log CFU/mL and a maximum of 6.2 log CFU/mL were detected in various farm environments, such as soil, feces, drinking water, boots, and milking machines. The finding of coliform in the milking machine is notable. Although the number of coliform bacteria did not exceed the hygiene standard in the study, the presence of bacteria indicated that microbial contamination through the milking machine is possible, suggesting that the hygiene management of the milking machine should not be neglected.

The growth of yeast and molds in milk is a common cause of spoilage in fermented dairy products [36]. The spoilage occurs because these microorganisms can grow well even at low pH. Yeasts and molds that spoil dairy products are generally acquired from the air in processing plants, manufacturing equipment, and the general environment (such as floors, drains, ventilation ducts, etc.) [37]. Our results showed that yeasts or molds were detected in various sites within the farm environment and processing plant. Notably, there were farms with very high pollution levels of 3.2~8.4 log CFU/100 cm^2^ in the milk filter. Molds form spores and may produce mycotoxins. In previous studies, two mycotoxins (aflatoxins and ochratoxins) were found in raw milk. Aflatoxin in raw milk is a stable, heat-resistant compound and is not completely destroyed by pasteurization [38]. Therefore, special attention is required for the sites with the highest contamination levels [39], and proper hygiene will be necessary to prevent contamination by yeasts or molds and produce safe and fresh milk.

Additionally, we found the presence of coliform in various sites of processing plants. In particular, some farms had coliforms above the standard value inside the vat and cheese mold, and 1.5 log CFU/mL was detected in the milking machine in one dairy farm. The milking machine comes in direct contact with the cow’s udder, and hence, it can directly contaminate the milk. Coliforms above the standard value were detected in 2 out of 8 farms for raw milk and 1 out of 6 farms for cheese. In general, coliform contamination of raw milk is related to feces, unclean udder and teats, the degree of cleaning of the milking container, hygiene of the milking environment, and contaminated water [40,41]. Therefore, in the case of farms with high coliform levels, it is recommended that proper hygiene management should be followed to prevent contamination from the aforementioned pollutant sources.

We also examined the differences in major microbial quality for each dairy farm. In the dairy farm environment, K and J farm showed higher levels of total aerobic bacteria compared to other dairy farms. In particularly, the K farm had the highest degree of contamination of total aerobic bacteria in the milk filter among the dairy farms, so it seems that hygiene management by disinfection or cleaning is necessary to reduce microbial contamination. In addition, the K farm presented to have the highest degree of contamination of yeasts and molds in the milk filter among dairy farms, so it is judged that the reuse of the disposable milk filter should be checked and corrected. The H farm had the highest level of coliform in boots among dairy farms, and care should be taken to ensure that the coliform does not spread to other spots in the dairy farm via regular cleaning of the boots. In dairy processing plants, the A and J farms had the highest levels of total aerobic bacteria. The J farm showed a high level of contamination in the vat bottom and drain hole, and the A farm showed a high level in the ripening room floor and ripening table bottom. In particular, the A farm showed a high level of 6.1 log coliform at the ripening table bottom, indicating that the contamination inside the ripening room was more severe than that of other dairy farms, and the counts of yeasts and molds were also the highest in the vat bottom and drain hole. As a result, the A farm showed higher overall microbial contamination in dairy processing plants compared to other dairy farms, and it seems that hygiene management for microbial contamination control is needed throughout the dairy processing plant.

*B. cereus* is a kind of soil bacteria that is widely distributed in the natural world and in foods, and most of the *B. cereus* found in pasteurized milk is due to soil contamination of the teat [42]. Additionally, *B. cereus* has heat resistance and a high spore production ability; when appropriate conditions are met, it proliferates vigorously and causes spoilage and deterioration, and it is thought to be detected at a higher level than other foodborne pathogens in dairy environments [43,44]. To date, the presence of *B. cereus* has long been a threat to the dairy industry and has been known as a major pathogen, causing food poisoning through milk and dairy products [7,27]. Our results showed a similar trend to the previous study [6,42]. In our research, 11.1% and 18.2% of the workers’ shoes and the bottom of the milking station in contact with the soil had *B. cereus* contamination.

*S. aureus* is a pathogen that causes mammary gland inflammation in the udder of dairy cows and results in great economic losses in the dairy industry worldwide. In this study, *S. aureus* was detected in only three milk filters in the dairy environment and none in the processing plant. The milk filter is an essential part of the milking process and is used to keep the milk clean by preventing debris and foreign substances from entering the bulk milk tank. According to previous studies, many cases of contamination of milk filters by pathogenic bacteria, such as *S. aureus*, *E. coli* O157, *Salmonella*, and *L. monocytogenes,* have been reported [45,46]. Similarly, our study results also showed the possibility of contamination of the milk filter by pathogenic bacteria. These results highlight the importance of single-use milk filters for preventing filter contamination and emphasize that we should check secondary contamination from workers to other sites.

We discovered *S. aureus* JDFM SA01 in the process of an experiment to detect and identify pathogenic bacteria in dairy farms and reported the whole genome sequence analysis of *S. aureus* strain JDFM SA01, isolated from a milk filter collected from a Korean dairy farm [13]. In this study, we performed a toxicity test and analyzed the biofilm formation characteristics using RNA-seq analysis to further analyze the molecular characteristics of *S. aureus* JDFM SA01. As the study of host-pathogen interactions and bacterial pathogenesis continues to increase, the *C. elegans* model offers advantages for studies of bacterial toxicity and host defense systems [47,48]. Therefore, *C. elegans* is a promising model for evaluating the toxicity of various pathogens [49]. The toxicity test results indicate that *S. aureus* JDFM SA01 is highly toxic to the *C. elegans* host and would be detrimental when ingested by the human host. Staphylococcal food poisoning is commonly associated with *S. aureus* contamination after pasteurization or the presence of *S. aureus*, which produces a large number of toxins in milk before pasteurization [50]. Many *S. aureus* strains are capable of producing extracellular protein toxins and virulence factors that contribute to pathogenicity, such as heat stable enterotoxins that can be active during and after pasteurization. [51]. In this study, *S. aureus* JDFM SA01 was detected in the raw milk and milking facilities; therefore, it may pose a potential risk of food poisoning.

Biofilm formation by *S. aureus* is an important issue in the dairy industry [11,52]. The biofilm-forming ability of *S. aureus* promotes adherence and colonization of microorganisms on milking equipment and dairy production facilities and resistance against antibiotics [53]. For this reason, we additionally confirmed the biofilm formation ability, and there was a risk of contamination in the dairy environment due to the biofilm formation ability of the *S. aureus* JDFM SA01 strain. Biofilms are potential sources of pathogenic bacteria for milk contamination in bulk tanks, so preventing biofilm formation in milking facilities is a very important step in producing safe, high-quality milk [52,53]. Several previous studies have shown the risk of biofilm formation of pathogenic bacteria in milking equipment [54,55]. Our results show that *S. aureus* isolated from milk filters has a high biofilm-forming ability. This finding suggests the possibility of formation and contamination by biofilms if milking facilities and bulk tanks, including milking machines, are contaminated by *S. aureus*.

Finally, we identified the correlation between the biofilm formation characteristics and gene expression of *S. aureus* JDFM SA01 through RNA-seq analysis. In biofilms, the genes encoding fibrinogen-binding protein fib, gamma-hemolysin components hlgA and hlgC, and virulence factors of *S. aureus* were significantly upregulated. The upregulation of genes encoding surface proteins fib, fnbA, can, and icaC and genes that promote the adhesion of JDFM SA01-induced biofilms (IsdA, IsdB, IsdC, and IsdH) might clarify the improved biofilm viability and biomass. Upon GO functional enrichment, 322, 111, and 336 specific GO terms in the biological process, cellular component, and molecular function were confirmed, respectively. These aspects showed that *S. aureus* JDFM SA01 could form stable and mature biofilms.

## 5. Conclusions

Until now, various microbial studies related to milk have been conducted in various countries, including Europe, Australia, and the United States, but related studies have been insufficient in Korea. Therefore, we investigated the prevalence and characterization of microorganisms in various spots in dairy farm environments and processing plants in Korea. Overall, it was confirmed that hygiene was relatively good across the farms, except for several spots, but raw milk always can be exposed to pathogenic bacteria and thus needs to be hygienically controlled, as it can cause food poisoning when contaminated. This study provides insight into the prevalence of hygiene indicator bacteria and pathogenic bacteria in Korean dairy farms. This work, along with a microbial study related to dairy farms, has laid the foundation for research to produce hygienic and high quality milk and dairy products.

## Figures and Tables

**Table 1:** Dairy farm environment and processing plant sample list.

	Samples	Number of Samples
Dairy farm environment	Milk filter, Boots, Milking floor, Milking machine, Soil and feces, Drinking water	171
Processing plant	Vat inside, Vat bottom, Cheese knife, Cheese mold, Processing room floor, Drain hole, Niche, Ripening room floor, Ripening table, Ripening table bottom, Cheese, Raw milk	273
	Total	444

**Table 2:** Mean (±SD) total aerobic bacteria counts in the environment of each dairy farm (A to K).

Sample	No. of Samples	Number of Microorganisms on Each Spot (Mean log CFU/mL or log CFU/100 cm^2^)										
		A	B	C	D	E	F	G	H	I	J	K
Soil and feces	27	8.7 ± 0.5	8.3 ± 0.2	- ^1^	7.4 ± 0.5	-	9.0 ± 0.1	8.5 ± 0.1	9.5 ± 0.1	6.6 ± 0.0	7.9 ± 0.2	7.8 ± 0.2
Drinking water	21	4.1 ± 0.3	3.8 ± 0.0	-	3.2 ± 0.0	-	5.8 ± 0.1	4.2 ± 0.1	4.1 ± 0.0	4.2 ± 0.1	-	-
Milking floor	27	4.2 ± 0.1	3.8 ± 0.2	-	4.3 ± 0.2	-	2.6 ± 0.9	2.1 ± 0.5	4.9 ± 0.1	4.3 ± 0.2	6.3 ± 0.7	9.0 ± 0.1
Milking machine	21	1.3 ± 0.1	ND ^2^	-	ND	-	4.5 ± 0.3	ND	-	-	4.5 ± 0.8	4.7 ± 0.9
Milk filter	18	6.1 ± 0.3	ND	-	3.4 ± 0.0	-	3.3 ± 0.4	ND	-	-	6.0 ± 0.1	9.2 ± 0.5
Boots	33	4.7 ± 0.6	5.4 ± 0.1	3.7 ± 0.8	4.1 ± 0.4	4.3 ± 0.1	3.9 ± 0.3	4.0 ± 0.2	5.3 ± 0.4	3.4 ± 0.0	8.8 ± 0.0	7.5 ± 0.1

^1^ -: Not tested. ^2^ ND: Not detected.

**Table 3:** Mean (±SD) total aerobic bacteria counts in the processing plants of each dairy farm (A to K).

Sample	No. of Samples	Number of Microorganisms on each Spot (Mean log CFU/mL or log CFU/100 cm^2^)										
		A	B	C	D	E	F	G	H	I	J	K
Vat inside	30	ND ^2^	4.2 ± 0.1	ND	- ^1^	ND	0.7 ± 0.9	0.5 ± 0.1	ND	ND	ND	ND
Vat bottom	30	5.3 ± 0.4	3.9 ± 0.2	5.5 ± 0.1	-	2.1 ± 0.1	4.8 ± 0.5	5.7 ± 0.1	4.7 ± 0.2	2.8 ± 0.1	8.3 ± 0.5	6.9 ± 0.3
Cheese knife	18	ND	ND	-	-	ND	ND	ND	-	-	ND	-
Cheese mold	21	ND	ND	-	-	ND	ND	ND	-	-	ND	ND
Processing room floor	30	6.4 ± 0.9	3.0 ± 0.1	2.8 ± 0.6	-	1.8 ± 0.5	4.9 ± 0.1	2.5 ± 0.5	ND	0.7 ± 0.0	5.2 ± 0.3	3.2 ± 0.2
Drain hole	30	4.8 ± 0.2	5.3 ± 0.0	4.3 ± 0.1	-	2.1 ± 0.9	4.5 ± 0.2	2.0 ± 0.5	1.4 ± 0.1	5.3 ± 0.2	7.5 ± 0.9	6.0 ± 1.9
Niche	27	3.8 ± 0.2	-	5.2 ± 1.3	-	2.2 ± 0.1	4.3 ± 0.7	4.6 ± 0.2	1.9 ± 0.0	2.5 ± 0.3	5.4 ± 0.1	5.8 ± 1.3
Ripening room floor	21	5.5 ± 0.9	ND	-	-	-	1.7 ± 0.1	0.5 ± 0.7	ND	2.0 ± 0.2	4.3 ± 0.4	-
Ripening table	24	-	ND	1.4 ± 0.8	-	-	1.4 ± 0.1	0.7 ± 0.1	4.6 ± 0.3	1.5 ± 0.3	2.0 ± 1.0	-
Ripening table bottom	24	6.9 ± 1.2	ND	5.4 ± 2.0	-	-	2.5 ± 0.3	1.7 ± 0.1	4.3 ± 0.1	0.7 ± 0.1	5.7 ± 1.8	-
Cheese	18	6.2 ± 0.6	5.4 ± 0.1	-	-	1.8 ± 0.6	2.1 ± 0.2	4.1 ± 0.1	-	-	8.0 ± 0.1	-
Raw milk	24	6.2 ± 0.5	4.1 ± 0.0	-	-	-	-	4.1 ± 0.1	5.1 ± 0.1	5.3 ± 0.3	5.9 ± 0.1	5.3 ± 0.7

^1^ -: Not tested. ^2^ ND: Not detected.

**Table 4:** Mean (±SD) psychrotrophic bacteria counts in the environment of each dairy farm (A to K).

Sample	No. of Samples	Number of Microorganisms on Each Spot (Mean log CFU/mL or log CFU/100 cm^2^)										
		A	B	C	D	E	F	G	H	I	J	K
Soil and feces	27	9.2 ± 0.7	8.2 ± 0.1	- ^1^	6.5 ± 0.1	-	9.0 ± 0.5	8.9 ± 0.0	7.9 ± 0.0	6.3 ± 0.1	6.0 ± 0.2	5.1 ± 0.2
Drinking water	21	5.2 ± 0.9	ND ^2^	-	2.7 ± 0.1	-	7.4 ± 0.1	4.5 ± 0.0	3.8 ± 0.0	4.4 ± 0.1	-	-
Milking floor	27	5.1 ± 0.1	5.4 ± 0.0	-	4.2 ± 0.1	-	3.5 ± 0.3	2.8 ± 0.1	2.8 ± 0.2	0.7 ± 0.0	5.0 ± 0.7	7.0 ± 0.1
Milking machine	21	ND	ND	-	ND	-	3.3 ± 0.1	ND	-	-	4.1 ± 0.8	2.1 ± 0.9
Milk filter	18	5.1 ± 0.3	ND	-	-	-	3.6 ± 0.7	ND	-	-	4.1 ± 0.1	7.1 ± 1.3
Boots	33	5.2 ± 0.5	6.0 ± 0.0	1.6 ± 1.4	3.9 ± 0.3	3.6 ± 0.1	3.9 ± 0.2	4.4 ± 0.0	5.1 ± 0.0	2.7 ± 0.5	6.0 ± 0.0	5.1 ± 0.1

^1^ -: Not tested. ^2^ ND: Not detected.

**Table 5:** Mean (±SD) psychrotrophic bacteria counts in the processing plants of each dairy farm (A to K).

Sample	No. of Samples	Number of Microorganisms on Each Spot (Mean log CFU/mL or log CFU/100 cm^2^)										
		A	B	C	D	E	F	G	H	I	J	K
Vat inside	30	ND ^2^	2.2 ± 0.1	ND	- ^1^	ND	ND	ND	ND	ND	ND	ND
Vat bottom	30	6.1 ± 0.2	1.5 ± 0.0	5.4 ± 0.2	-	1.7 ± 0.0	5.0 ± 1.0	5.6 ± 0.0	3.6 ± 0.0	2.4 ± 0.2	7.2 ± 0.5	4.1 ± 0.3
Cheese knife	18	ND	ND	-	-	ND	ND	ND	-	-	ND	-
Cheese mold	21	4.1 ± 0.6	ND	-	-	ND	ND	ND	-	-	ND	-
Processing room floor	30	4.2 ± 0.2	ND	3.9 ± 0.7	-	2.2 ± 0.1	5.2 ± 0.1	2.3 ± 0.0	ND	0.7 ± 0.1	4.0 ± 0.3	3.2 ± 0.2
Drain hole	30	3.8 ± 0.5	3.8 ± 0.1	3.8 ± 0.6	-	3.1 ± 0.1	5.8 ± 1.5	1.6 ± 0.5	ND	5.6 ± 0.4	5.0 ± 0.9	5.1 ± 2.9
Niche	27	2.8 ± 0.2	-	4.2 ± 1.3	-	4.4 ± 0.1	3.9 ± 0.2	5.8 ± 0.3	2.2 ± 0.1	2.2 ± 0.3	4.0 ± 0.1	5.1 ± 1.3
Ripening room floor	21	5.2 ± 0.2	ND	-	-	-	1.2 ± 0.2	0.5 ± 0.1	ND	1.5 ± 0.2	4.3 ± 0.4	-
Ripening table	24	ND	ND	ND	-	-	ND	2.1 ± 0.4	1.1 ± 0.5	1.4 ± 0.1	1.5 ± 0.1	-
Ripening table bottom	24	5.3 ± 0.4	ND	4.0 ± 0.4	-	-	1.5 ± 2.1	0.7 ± 0.0	ND	ND	5.1 ± 1.8	-
Cheese	18	5.1 ± 0.4	ND	-	-	3.0 ± 0.6	3.2 ± 0.2	4.0 ± 0.1	-	-	6.0 ± 0.7	-
Raw milk	24	4.2 ± 0.5	4.1 ± 0.0	-	2.7 ± 0.1	-	-	4.7 ± 0.1	4.1 ± 0.1	4.9 ± 0.4	5.0 ± 0.1	4.1 ± 0.7

^1^ -: Not tested; ^2^ ND: Not detected.

**Table 6:** Mean (±SD) yeast/mold counts in the environment of each dairy farm (A to K).

Sample	No. of Samples	Number of Microorganisms on Each Spot (Mean log CFU/mL or log CFU/100 cm^2^)										
		A	B	C	D	E	F	G	H	I	J	K
Soil and feces	27	4.2 ± 0.1	5.1 ± 0.1	- ^1^	2.3 ± 0.0	-	6.5 ± 0.0	4.5 ± 0.2	5.3 ± 0.0	3.1 ± 0.1	6.7 ± 0.0	4.3 ± 0.1
Drinking water	21	ND ^2^	ND	-	ND	-	3.8 ± 0.1	3.1 ± 0.0	ND	2.2 ± 0.2	-	-
Milking floor	27	ND	3.9 ± 0.0	-	ND	-	2.8 ± 0.5	ND	2.9 ± 0.2	4.8 ± 0.1	2.3 ± 0.3	3.6 ± 0.2
Milking machine	21	ND	ND	-	ND	-	4.0 ± 0.0	2.8 ± 0.1	-	-	1.2 ± 0.2	ND
Milk filter	18	5.8 ± 0.5	ND	-	-	-	ND	ND	-	-	3.2 ± 0.0	8.4 ± 0.2
Boots	33	4.8 ± 0.1	5.1 ± 0.1	4.3 ± 0.7	ND	2.7 ± 0.1	2.9 ± 0.0	2.7 ± 0.1	3.5 ± 0.4	3.9 ± 0.3	3.9 ± 0.1	1.5 ± 0.1

^1^ -: Not tested. ^2^ ND: Not detected.

**Table 7:** Mean (±SD) yeast/mold counts in the processing plants of each dairy farm (A to K).

Sample	No. of Samples	Number of Microorganisms on Each Spot (Mean log CFU/mL or log CFU/100 cm^2^)										
		A	B	C	D	E	F	G	H	I	J	K
Vat inside	30	ND ^2^	3.4 ± 0.0	ND	- ^1^	ND	0.5 ± 0.7	ND	ND	ND	2.4 ± 0.1	ND
Vat bottom	30	6.1 ± 0.9	ND	2.0 ± 0.8	-	2.1 ± 0.9	3.1 ± 0.6	2.4 ± 0.2	3.7 ± 0.1	4.0 ± 0.1	5.2 ± 0.4	5.2 ± 0.7
Cheese knife	18	ND	3.4 ± 0.2	-	-	ND	0.5 ± 0.7	ND	-	-	ND	-
Cheese mold	21	ND	3.4 ± 0.0	ND	-	ND	0.5 ± 0.7	ND	ND	ND	2.4 ± 0.1	ND
Processing room floor	30	4.4 ± 0.9	ND	4.6 ± 1.5	-	1.2 ± 0.8	ND	2.6 ± 0.4	ND	ND	3.0 ± 0.1	1.2 ± 0.2
Drain hole	30	6.8 ± 0.2	ND	3.4 ± 0.6	-	ND	4.0 ± 0.7	1.6 ± 0.2	1.8 ± 0.1	3.8 ± 0.2	4.4 ± 0.7	3.2 ± 1.2
Niche	27	3.8 ± 0.2	-	4.2 ± 1.1	-	1.8 ± 0.5	1.0 ± 0.1	1.4 ± 0.1	2.1 ± 0.2	4.7 ± 0.4	4.8 ± 0.0	2.8 ± 0.3
Ripening room floor	21	3.4 ± 0.2	ND	-	-	-	1.2 ± 0.1	3.0 ± 0.4	ND	ND	4.3 ± 0.0	-
Ripening table	24	3.5 ± 0.3	ND	3.5 ± 0.5	-	-	0.5 ± 0.1	4.1 ± 0.2	3.5 ± 0.2	ND	4.0 ± 0.6	-
Ripening table bottom	24	4.5 ± 0.2	ND	4.4 ± 1.6	-	-	ND	3.5 ± 0.6	5.5 ± 0.2	ND	3.9 ± 1.1	-
Cheese	18	6.2 ± 0.1	ND	-	-	ND	2.6 ± 0.8	3.6 ± 0.4	-	-	4.5 ± 0.1	-
Raw milk	24	2.1 ± 0.1	ND	-	3.4 ± 0.0	-	-	ND	3.0 ± 0.3	2.1 ± 0.2	3.8 ± 0.1	1.5 ± 0.5

^1^ -: Not tested. ^2^ ND: Not detected.

**Table 8:** Mean (±SD) coliform counts in the environment of each dairy farm (A to K).

Sample	No. of Samples	Number of Microorganisms on Each Spot (Mean log CFU/mL or log CFU/100 cm^2^)										
		A	B	C	D	E	F	G	H	I	J	K
Soil and feces	27	5.5 ± 0.2	ND ^2^	- ^1^	ND	-	5.3 ± 0.2	6.2 ± 0.0	3.8 ± 0.0	ND	ND	ND
Drinking water	21	1.5 ± 0.2	2.0 ± 0.2	-	ND	-	2.8 ± 0.1	ND	2.2 ± 0.2	ND	-	-
Milking floor	27	1.3 ± 0.6	1.7 ± 0.3	-	1.3 ± 0.4	-	ND	ND	2.6 ± 0.1	ND	3.4 ± 0.4	ND
Milking machine	21	ND	ND	-	ND	-	ND	ND	-	-	ND	1.5 ± 0.5
Milk filter	18	ND	ND	-	-	-	ND	ND	-	-	1.4	ND
Boots	33	2.4 ± 0.3	2.7 ± 0.1	ND	ND	1.6 ± 0.8	ND	2.8 ± 0.2	4.5 ± 0.3	ND	ND	2.1 ± 0.1

(1) -: Not tested. (2) ND: Not detected.

**Table 9:** Mean (±SD) coliform counts in the processing plants of each dairy farm (A to K).

Sample	No. of Samples	Number of Microorganisms on Each Spot (Mean log CFU/mL or log CFU/100 cm^2^)										
		A	B	C	D	E	F	G	H	I	J	K
Vat inside	30	ND ^2^	3.4 ± 0.0	ND	- ^1^	ND	ND	ND	ND	ND	ND	ND
Vat bottom	30	ND	ND	ND	-	3.1 ± 0.9	ND	ND	2.8 ± 0.5	ND	2.2 ± 0.5	2.0 ± 0.1
Cheese knife	18	ND	ND	-	-	ND	ND	ND	-	-	ND	-
Cheese mold	21	3.2 ± 0.2	ND	-	-	ND	ND	ND	-	-	ND	-
Processing room floor	30	5.3 ± 0.9	ND	ND	-	2.7 ± 0.1	ND	1.6 ± 0.4	ND	ND	2.1 ± 0.0	ND
Drain hole	30	ND	ND	ND	-	2.8 ± 0.1	2.9 ± 1.3	1.8 ± 0.2	ND	1.8 ± 0.4	ND	ND
Niche	27	ND	-	ND	-	1.7 ± 0.5	ND	ND	ND	ND	ND	1.7 ± 0.7
Ripening room floor	21	4.1 ± 1.0	ND	-	-	-	ND	ND	ND	ND	ND	-
Ripening table	24	ND	ND	ND	-	-	ND	ND	ND	ND	ND	-
Ripening table bottom	24	6.1 ± 1.2	ND	4.2 ± 0.4	-	-	ND	ND	ND	ND	ND	-
Cheese	18	2.3 ± 0.1	3.2 ± 0.1	-	-	1.8 ± 0.9	2.2 ± 0.1	1.0 ± 0.0	-	-	ND	-
Raw milk	24	2.3 ± 0.1	0.8 ± 1.1	-	ND	-	-	ND	2.6 ± 0.2	3.8 ± 0.1	3.8 ± 0.1	ND

^1^ -: Not tested. ^2^ ND: Not detected.

**Table 10:** Prevalence of *B. cereus* and *S. aureus* on each sample type.

Sample		No. of Samples	Isolations of Pathogen from Each Site	
			*Bacillus cereus*	*Staphylococcus aureus*
Processing plant	Vat inside	30	-	-
	Vat bottom	30	-	-
	Cheese knife	18	-	-
	Cheese mold	21	-	-
	Processing room floor	30	-	-
	Drain hole	30	-	-
	Niche	27	-	-
	Ripening room floor	21	-	-
	Ripening table	24	-	-
	Ripening table bottom	24	-	-
	Cheese	18	-	-
	Raw milk	24	-	-
Dairy farm environment	Milk filter	18	-	3
	Boots	33	6	-
	Milking floor	27	3	-
	Milking machine	21	-	-
	Soil and feces	27	5	-
	Drinking water	21	-	-

Note: Five pathogens (*E. coli* O157, *L. monocytogenes*, *C. perfringens*, *Salmonella* spp., and *Campylobacter* spp.) were not detected in samples (data not shown).

**Table 11:** Prevalence of *B. cereus* and *S. aureus* on each dairy farm (A to K).

Farm	Isolations of each Pathogen on Each Farm		Total Number of Pathogens per Farm
	*Bacillus cereus* (*n* = 14)	*Staphylococcus aureus* (*n* = 3)	
A	2	3	8
B	2	0	2
C	0	0	0
D	5	0	5
E	3	0	3
F	0	0	0
G	0	0	0
H	0	0	0
I	0	0	0
J	2	0	2
K	0	0	0

Note: Other pathogens (*E. coli* O157, *L. monocytogenes*, *C. perfringens*, *Salmonella* spp., and *Campylobacter* spp.) were not detected in dairy farms (data not shown).

**Table 12:** Differentially expressed genes in biofilms compared with planktonic biofilms.

ID	log2 (Fold_Change)	logCPM	*p*-Value	Product	Gene Name
*S-aureus*_1_00146_gene	73.927580	7.595026	1.5745E-100	Fibrinogen-binding protein	Fib
*S-aureus*_1_00142_gene	34.971355	5.156831	4.87239E-53	Staphylococcal complement inhibitor	Scn
*S-aureus*_1_01520_gene	28.372963	6.429903	1.13995E-65	Gamma-hemolysin component A	HlgA
*S-aureus*_1_00168_gene	14.364441	3.082528	2.50483E-19	Iron-regulated surface determinant protein C	IsdC
*S-aureus*_1_01444_gene	4.896297	7.553409	9.02819E-24	Fibronectin-binding protein A	FnbA
*S-aureus*_1_00170_gene	4.302082	3.382092	1.62205E-10	Iron-regulated surface determinant protein B	IsdB
*S-aureus*_1_02174_gene	3.507874	6.374902	8.07925E-15	Iron-regulated surface determinant protein H	IsdH
*S-aureus*_1_00169_gene	2.751611	4.668524	2.26694E-08	Iron-regulated surface determinant protein A	IsdA
*S-aureus*_1_01278_gene	2.663345	0.792624	0.053006961	putative poly-beta-1,6-N-acetyl-D-glucosamine export protein	IcaC
*S-aureus*_1_01256_gene	2.482370	7.790885	3.16301E-09	Collagen adhesin	Can
*S-aureus*_1_01519_gene	2.166117	6.050228	1.89091E-06	Gamma-hemolysin component C	HlgC

## Data Availability

The data presented in this research can be used upon request with the corresponding author.
